# Student Life in Germany

**Published:** 1870-03

**Authors:** 


### STUDENT LIFE IN GERMANY.

A YOUNG lad writes from Heidelberg, where there are about a thousand students from all parts of the world: "The best swordsman in the university is an American, from Cincinnati, Ohio; he looks like a big savage, because he drinks so much beer. Some students drink twenty-four chopin (pints) in one evening. When they are on a spree, they all stand up at twelve o'clock, and at each stroke of the clock each man drinks a glass containing one pint. But only a few can do this. During the evening, if a student has taken as much beer as he can, and is not ready to go home, he goes out, throws up what he has taken, and commences again. Such are the stories of the German people, who look up to the students as if they were something extra."

We may add ourselves that we saw many students with cheeks disfigured by an ugly scar, to be carried through life, having been made by a sword's thrust in a duel, such a disfigurement being considered as proof of heroism, and yet American youths crowd German universities to finish their education!

